# From transcriptomics to digital twins of organ function

**DOI:** 10.3389/fcell.2024.1240384

**Published:** 2024-06-26

**Authors:** Jens Hansen, Abhinav R. Jain, Philip Nenov, Peter N. Robinson, Ravi Iyengar

**Affiliations:** ^1^ Department of Pharmacological Science and Institute for Systems Biomedicine, Icahn School of Medicine at Mount Sinai, New York, NY, United States; ^2^ College of Arts and Sciences, University of Pennsylvania, Philadelphia, PA, United States; ^3^ Berlin Institute of Health at Charité Rahel Hirsch Center for Translational Medicine, Berlin, Germany

**Keywords:** digital twin, dynamical modeling, systems biology, networks, transcriptomics

## Abstract

Cell level functions underlie tissue and organ physiology. Gene expression patterns offer extensive views of the pathways and processes within and between cells. Single cell transcriptomics provides detailed information on gene expression within cells, cell types, subtypes and their relative proportions in organs. Functional pathways can be scalably connected to physiological functions at the cell and organ levels. Integrating experimentally obtained gene expression patterns with prior knowledge of pathway interactions enables identification of networks underlying whole cell functions such as growth, contractility, and secretion. These pathways can be computationally modeled using differential equations to simulate cell and organ physiological dynamics regulated by gene expression changes. Such computational systems can be thought of as parts of digital twins of organs. Digital twins, at the core, need computational models that represent in detail and simulate how dynamics of pathways and networks give rise to whole cell level physiological functions. Integration of transcriptomic responses and numerical simulations could simulate and predict whole cell functional outputs from transcriptomic data. We developed a computational pipeline that integrates gene expression timelines and systems of coupled differential equations to generate cell-type selective dynamical models. We tested our integrative algorithm on the eicosanoid biosynthesis network in macrophages. Converting transcriptomic changes to a dynamical model allowed us to predict dynamics of prostaglandin and thromboxane synthesis and secretion by macrophages that matched published lipidomics data obtained in the same experiments. Integration of cell-level system biology simulations with genomic and clinical data using a knowledge graph framework will allow us to create explicit predictive models that mechanistically link genomic determinants to organ function. Such integration requires a multi-domain ontological framework to connect genomic determinants to gene expression and cell pathways and functions to organ level phenotypes in healthy and diseased states. These integrated scalable models of tissues and organs as accurate digital twins predict health and disease states for precision medicine.

## Introduction

Accurate multiscale computational models of physiological functions of different organs within the human body have the potential to revolutionize our understanding of human biology and greatly advance the practice of medicine. Vast amounts of data are being collected in different domains of genomics, biochemistry, cell biology and physiology and clinical sciences. It will be necessary to bring together these data to understand the physiology of organ systems. Physiology is dynamics (Rubin et al., 2019). Understanding how the function of organs changes over time is essential for understanding both homeostasis for health and disease origins and progression. The functions of organs arise from cell-level physiological activity. Examples include the heart, where ability of cardiomyocytes to contract in a rhythmic and coordinated fashion underlie the beating of heart, and the kidney where ability of different cell types of the nephron to filter large molecules and reabsorb ions, water and small molecules underlie our ability to regulate water balance, excrete end products of metabolism, maintain pH balance in blood, and control blood pressure. Thus, to generate accurate predictive models of organ function, the first step is to build accurate models of whole cell functions. Such models should consider the key components and pathways within the cell; the networks that arise from interactions between pathways and pathway components; the topological features of the networks including the feedback loops, feedforward loops and bifans (Milo et al., 2002) which enable processing of information within the cell (Ma’ayan et al., 2005); and state changes driven by bistable switches (Bhalla and Iyengar, 1999; Tanaka and Augustine, 2008).

To go from cell-based models to organ level models we need to consider how the different cell types in the organ function and interact as well as the role of the extracellular matrix in controlling the mechanical and signaling properties of the organ. Multiple anatomical structures make up each organ. Blood vessels are one example of tissue components contributing to an organ’s physiology. Blood vessels have vascular smooth muscle cells, fibroblasts, endothelial cells (Sturtzel, 2017) that line the wall of the blood vessels and make up the capillaries, as well as pericytes (Lee and Chintalgattu, 2019) in some organs. The latter two cell types are often the source of important signaling molecules and sense mechanical forces such as the pressure from blood flow to control organ function.

Changes in cell state are driven by changes in gene expression patterns that control whole cell responses. Transcriptomic profiles represent cell identity as well as cell state. Hence, we hypothesize that changes in gene expression patterns can be used to predict dynamic physiological capabilities. We describe our initial approach to test this hypothesis and provide preliminary evidence that the approach we propose could work. Our approach consists of two sets of operations that integrate two different modeling approaches. First, we take a ranked list of genes, typically differentially expressed mRNAs indicative of two different conditions (states) the cells or organs are in and create networks using pathway information from prior knowledge databases. These interacting pathways are enriched for the differentially expressed genes and could account for change in activity. Going from genes to pathways using prior knowledge is a very widely used statistical modeling approach called gene-set enrichment analysis (Subramanian et al., 2005). Second, the reactions participating in identified pathways that together make up edges in directed subgraphs or graphs are readily converted to systems of coupled differential equations. These systems of coupled differential equations are dynamical models that can be used to run simulations to predict how cell biochemical or physiological functions change with time. Here, we describe how this two-step algorithm can work, and eventually become part of a larger algorithm for a digital twin. In biology, digital twins can be thought of multi-scale computational models that can predict physiological events from genomic and molecular data. Such predictions may be at the cell level, tissue/organ level or at the whole organism level. In this review we consider the cell and organ levels.

## Computational approaches to modeling dynamics

To support widespread use of single cell transcriptomics multiple approaches to conduct trajectory analyses from time series and single timepoint experiments have been published, and these approaches are described and compared in a review article (Ding et al., 2022). This approach has been particularly useful in mapping trajectories during developmental processes and provide useful insight into precursor and differentiated cell types in many organ systems. However, all these approaches provide pseudo-time series outputs that can only be constrained by experimental time series analyses. Pseudo time series order entities with respect to one another to infer trajectories. For example, ligand activation of receptor and stimulation of membrane effectors occur prior to activation of protein kinases. This information can be used to develop trajectories from receptors to physiological effectors such as channels and metabolic enzymes. Pseudo time series analyses do have value in understanding the progression of biological states and we had used pseudo time series in a 2005 study (Ma’ayan et al., 2005) to understand the role of regulatory motifs such as feedforward and feedback loops in signal propagation from receptor to transcription factors to control the duration of transcription factor activation. Orthogonal experimental approaches such as single nucleus ATAC Seq and CRISPR/Cas9 mediated gene modification provide mechanistic insights into trajectory analyses and together they may help define realistic time-dependent predictions in the future. The limitation of pseudo-time series to capture physiological dynamics lies in its inability to be scalable and hence is likely to be of limited value in realistic digital twins.

A combination of proteomic and phenotypic feature measurements to identify new drug combinations that would work on drug resistant cells uses differential equation-based modeling to develop predicted responses of cancer cells (Frohlich et al., 2018). The approach is similar to the PK/PD modeling widely used in pharmacology that is a mainstay in the drug discovery process. Such approaches that integrate perturbation data with prior pathway information can predict drug responses, especially responses to combination therapy. The Cell Box Software suite (Yuan et al., 2021) provides a useful tool set for such analyses including network development in a purely data driven manner. Limitations of such a modeling approach is that the captured perturbation dynamics depend on many undefined reactions and rate constants and hence it is uncertain whether such an approach will work under different physiological states and conditions without specific large-scale gathering of experimental data for each condition.

An integrative dynamical model using coupled differential equations that are solved in a standard solver using MATLAB has been developed to predict macrophage polarization (Zhao et al., 2021; Zhao and Popel, 2021). The scope of the model is extensive and impressive, although surprisingly the prostaglandin biosynthesis and signaling pathways are missing. Nevertheless, the model represents an important step in the development of the virtual macrophage that can predict macrophage polarization and functions in various physiological states. Such models could well be adapted to describe other types of blood cells although and their trajectories in health and disease. Beyond cell level models, these researchers have proposed approaches that integrate omics data and dynamical models for tissue level angiogenesis models that represent communication between different cell types (Zhang et al., 2022). Such approaches are likely to be useful in developing digital twins for angiogenesis.

The approach we propose here has some similarities and differences with these previously described models. Our approach is focused on getting the cell-level molecular and pathway details “right” and then determining if dynamical models based on granular biochemical and biophysical reactions can be used to predict and understand physiological behaviors at the cell level and at the organ level. The pros and cons of this approach and its use as the core of digital twins of organs are discussed below.

## Advantages and challenges in the use of numerical analyses to predict physiological dynamics

Modeling biochemical and physiological processes using standard chemical kinetics is better than most other approaches because this is the most realistic representation of these processes including those involved in generation and sensing of forces. We have long favored the use of chemical kinetics representations and shown that we can make non-intuitive experimentally verifiable predictions. Our model using systems of ordinary differential equations (Bhalla and Iyengar, 1999) that predicted the existence bistable positive feedback loops that can enable switching cellular states has been experimentally validated by others in cerebellar long-term depression (Tanaka and Augustine, 2008). Our spatial partial differential equation model predicting selective cAMP accumulation in dendrites as compared to cell body of neurons (Neves et al., 2008) was validated using a cAMP biosensor in mouse brain slice tissues by Castro et al. (2010). We have continued to use this approach to develop predictive models of interactions between subcellular processes. We predicted that dynamic balance between membrane vesicle transport and microtubule growth is required for neurite outgrowth (Yadaw et al., 2019). We used gene knockdown of vesicle transport and docking protein to demonstrate the validity of our prediction (Yadaw et al., 2019; Hansen et al., 2022). Despite these successes, challenges have always been present. Initially some of the challenges were computational, such as computational costs and propagation of errors. With the exponential increase in computational capability these challenges have become less of a barrier. However, the biological challenges persist. The cellular concentrations of most proteins have yet to be explicitly measured in most cell types of the human body, although it is often possible to estimate or guesstimate them from the vast biochemistry and cell biology literature. Also, reaction rates are often not known. Databases such as BRENDA (Schomburg et al., 2017) are useful, although kinetic information regarding mammalian systems is limited. Another useful resource is Bionumbers which contains many “average” values used to set up the models for numerical simulations (Milo et al., 2010).

## Gene expression changes to neurite outgrowth, a whole cell response: identifying and modeling cell regulatory pathways and networks

**FIGURE 1:**
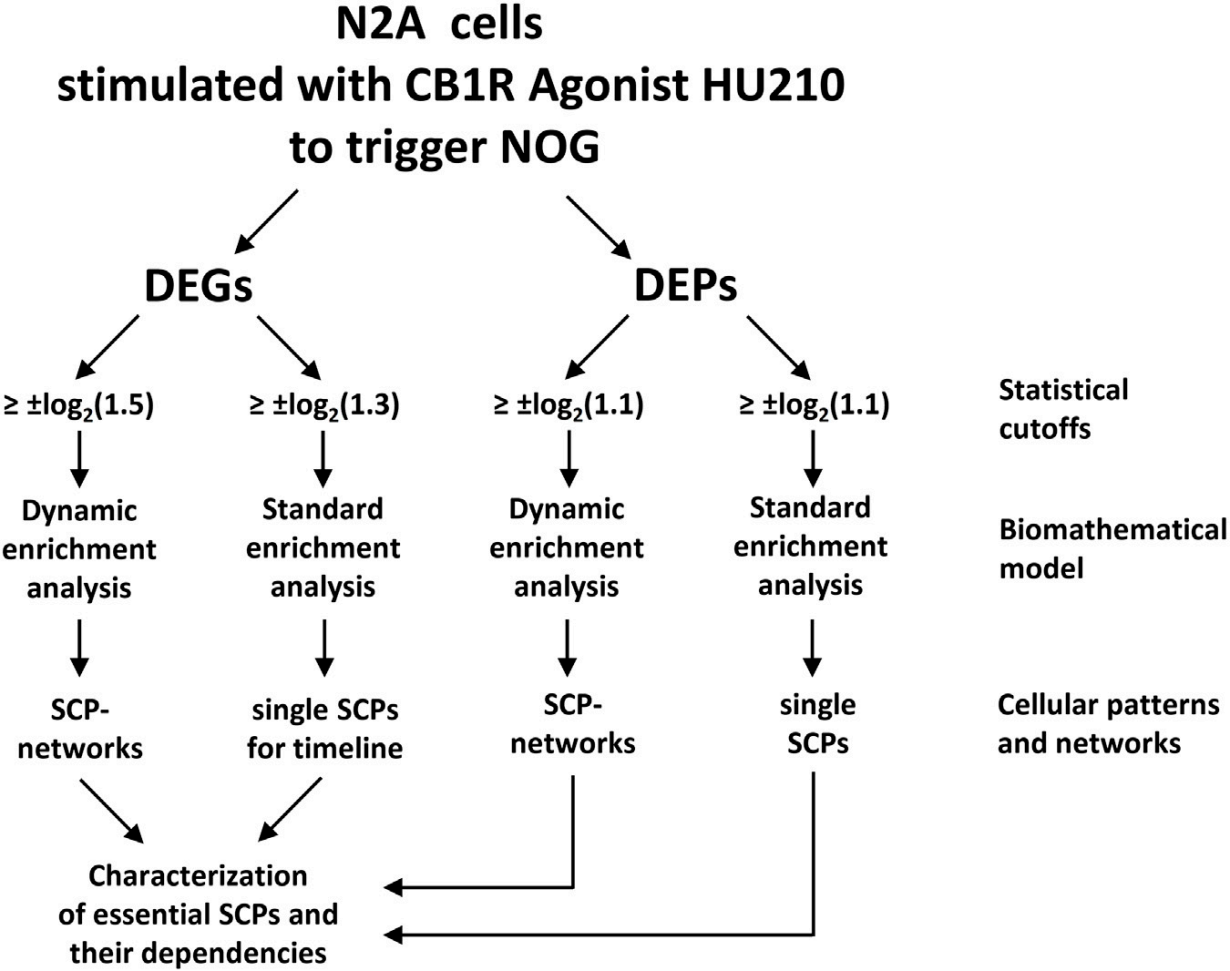
A flow chart showing the steps used for building networks of subcellular pathways (SCPs) underlying neurite outgrowth (NOG). Standard and dynamic enrichment analysis refer to methods used inferring pathway from differentially expressed genes (DEGs) or proteins (DEPs). Reproduced from and for details see Hansen et al. (2022).

In a recent study, we have shown how transcriptional patterns can be used by cells to drive cell state changes and whole cell responses to external signals through well-known canonical pathways (Hansen et al., 2022). Although our study is based on bulk transcriptomics and discovery proteomics obtained from only one cell line cultured in isolation, our analysis strategy should be applicable to single cell transcriptomics and other omics technologies as well. Briefly, we treated the neuronal cell line Neuro2A (N2A) with an agonist for the cannabinoid receptor 1 (CB1R) to induce neurite outgrowth. Differentially expressed genes and proteins induced after different stimulation periods were subjected to pathway enrichment analysis (Figure 1), using the Molecular Biology of the Cell Ontology (MBCO), a cell biology focused ontology that was generated in our lab (Hansen et al., 2017).

**FIGURE 2:**
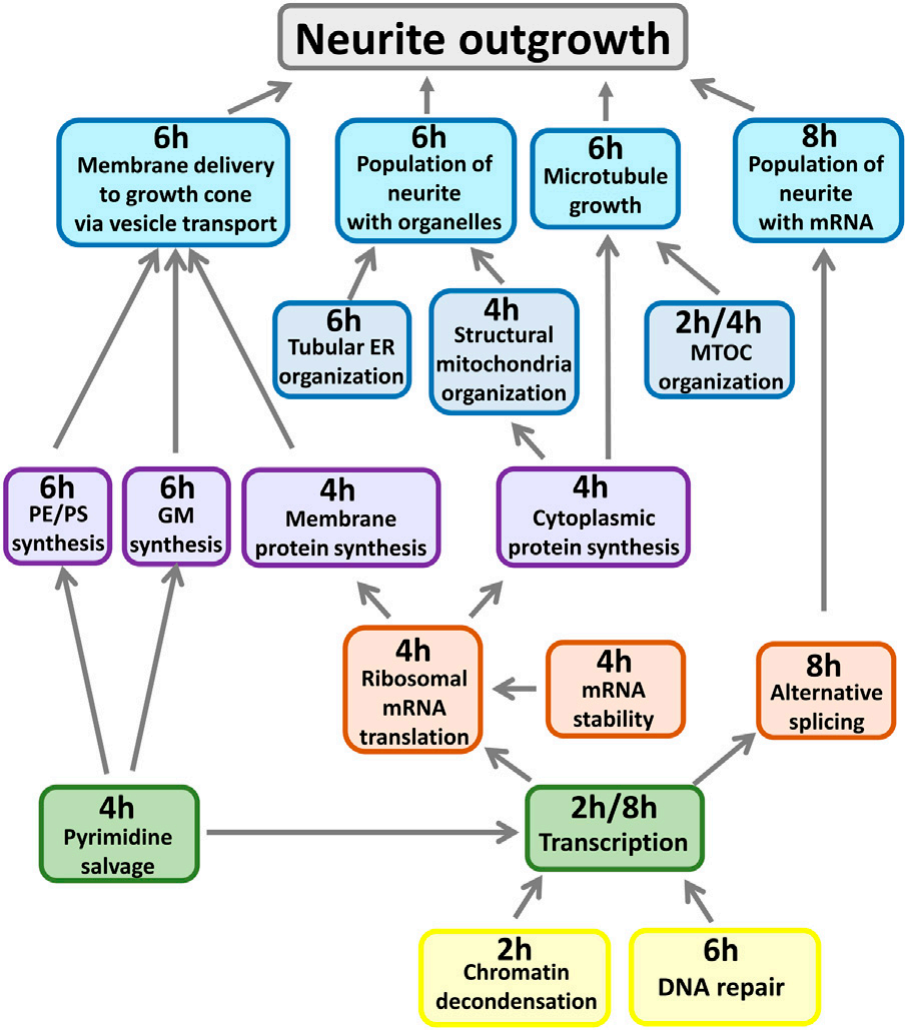
Whole cell response requires deep and distributed responses. All shown SCPs are related to growth of the neurite shaft or scaffold. Described functions summarize pathway activities predicted at indicated time points from gene expression profiles induced by CB1R stimulation. MTOC: Microtubule organization center, PE/PS: Phosphatidylethanolamine/-choline, GM: Ganglioside, ER: Endoplasmic reticulum. Adapted from and for details see Hansen et al. (2022).

**FIGURE 3:**
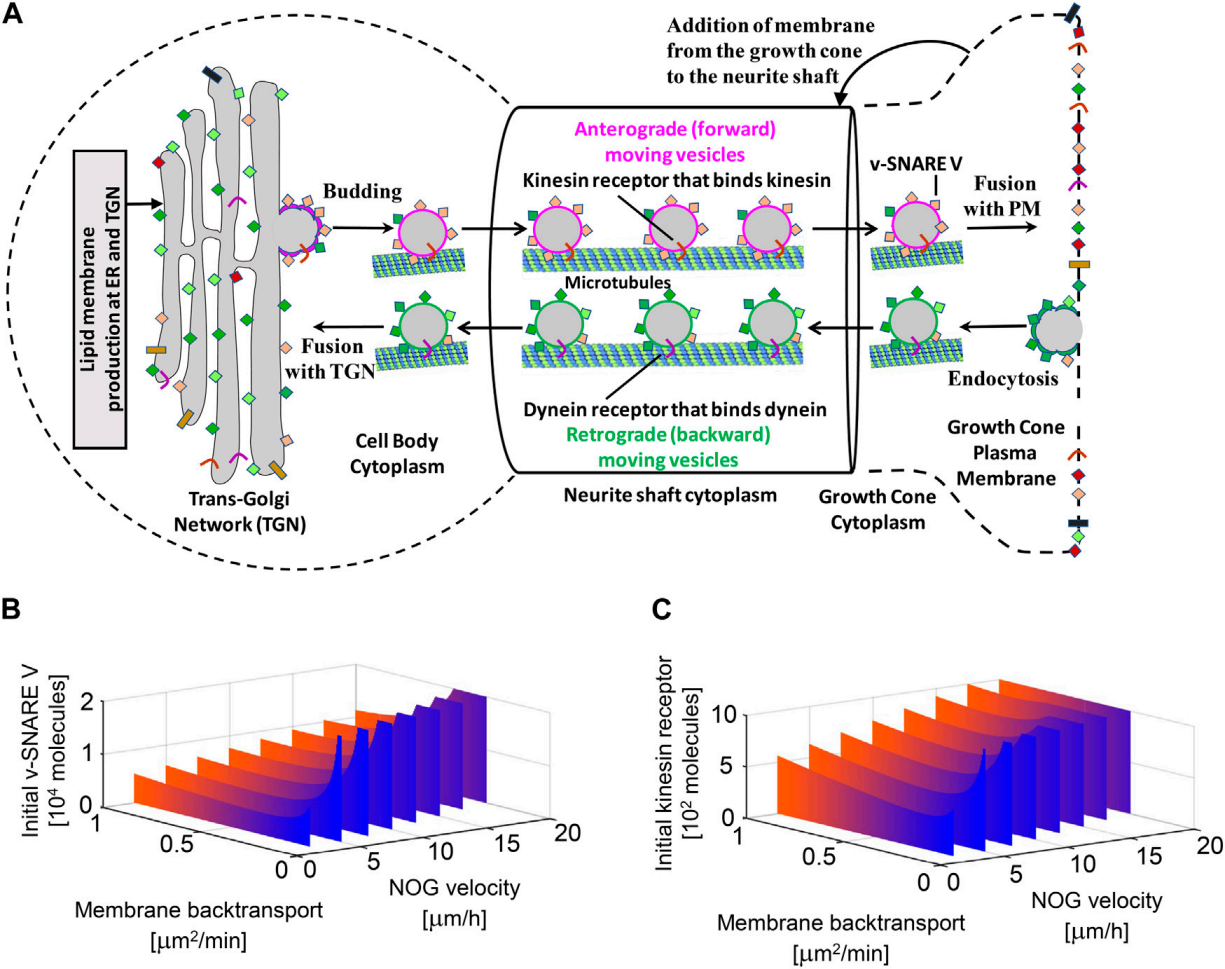
Multicompartment ODE model showing the necessity for vesicle recycling (i.e., membrane back transport) for neurite growth. Recycling is required to maintain the dynamic concentrations of the motor protein kinesin and the fusion protein v-SNARE for the whole cell response. TGN, Trans-Golgi Network; PM, Plasma Membrane. Reproduced from Hansen et al. (2022). For details see Hansen et al. (2022) and Yadaw et al. (2019).

We identified many subcellular processes (SCPs), which are commonly thought of as constitutive pathways that are operational in many, if not all cell types. While these SCPs such as alternative splicing, pyrimidine salvage and membrane protein synthesis are universal, the ability of the extracellular signal to regulate them in a coordinated manner gives the cell additional capacity to mount the whole cell response. Our data documents that the canonical SCPs are activated in a chronological order that matches their dependencies (Figure 2). It can be readily seen that many cellular pathways in different organelles such as the nucleus, endoplasmic reticulum (ER), cytosol and growing neurite compartments are involved. Although shown in an abstracted form for clarity, each of the SCPs shown in Figure 2 contains multiple interacting proteins that come together to form larger functional networks. The different pathways must work in a highly coordinated fashion and imbalances in their coordination can lead to stoppage of the cellular responses. This conclusion is supported by dynamical modeling of one set of SCPs involved in transporting newly synthesized membranes as cytosolic vesicles from the Trans-Golgi network (TGN) through the neurite shaft to the growing tip at the end of the neurite. The new membrane is needed to build the axonal shaft as neurite grows. The importance of dynamics is inferred from the multicompartment ordinary differential equation (ODE) model that simulates the movement of newly synthesized vesicles from the TGN in the cell body to the growing tip. After developing an analytical solution for the prediction of parameter settings that allow neurite outgrowth at a given velocity and literature-curated model constraints with high accuracy, we could show how multiple pathways interact with each other to generate the whole cell response. Our analysis revealed that increased neurite outgrowth depends on increased backward vesicle traffic from the neurite tip to the TGN (Figure 3). This initially counter intuitive dependency ensures back transport of components needed for forward vesicle traffic. Such focused simulations within the larger overall computational model are likely to be critical parts for verification of the underlying pathways and validation of mechanisms at the subcellular levels could also be used to parameterize and identify the uncertainty in how interactions between SCP subnetworks as well as interactions with the cell and extracellular matrix lead to dynamics of organ level functions.

## Predicting cardiomyocyte electrophysiology and contractility from transcriptomic changes

The ability to develop a dynamical model for cell functions is dependent on the pathways and networks inferred from the DEGs and DEPs. Once these networks are identified, pathway activities can be readily connected to systems of coupled differential equations that can be used for multi-compartment ODE models or PDE models. Although most models that capture biochemical SCPs use a pathways framework, biophysical models can also capture changes in gene expression to predict responses to perturbation. In a recent study using cardiomyocytes differentiated from healthy human subjects, gene expression changes induced by tyrosine kinase inhibitor drugs that are effective cancer therapeutics was used to develop computational models that predict arrhythmogenic responses to cancer drug therapy in individuals (Shim et al., 2023). Changes in levels of gene expression of different channel proteins by drugs were scaled and incorporated as changes in level of channel proteins into a multicompartment ODE model of cardiomyocyte action potential and contractility. Experimental measurements of cardiomyocyte action potentials, intracellular calcium, and contraction in the cardiomyocytes demonstrated that modeling predictions were mostly (80%) accurate. The simulations were also able to predict responses to drugs and a second perturbation such as hypokalemia (low potassium). Together the biochemical and biophysical models demonstrate the ability of numerical simulations to use transcriptomic data for predictions.

## An integrated algorithm to go from differentially expressed genes to biochemical dynamics: eicosanoid biosynthesis network in macrophages

**FIGURE 4:**
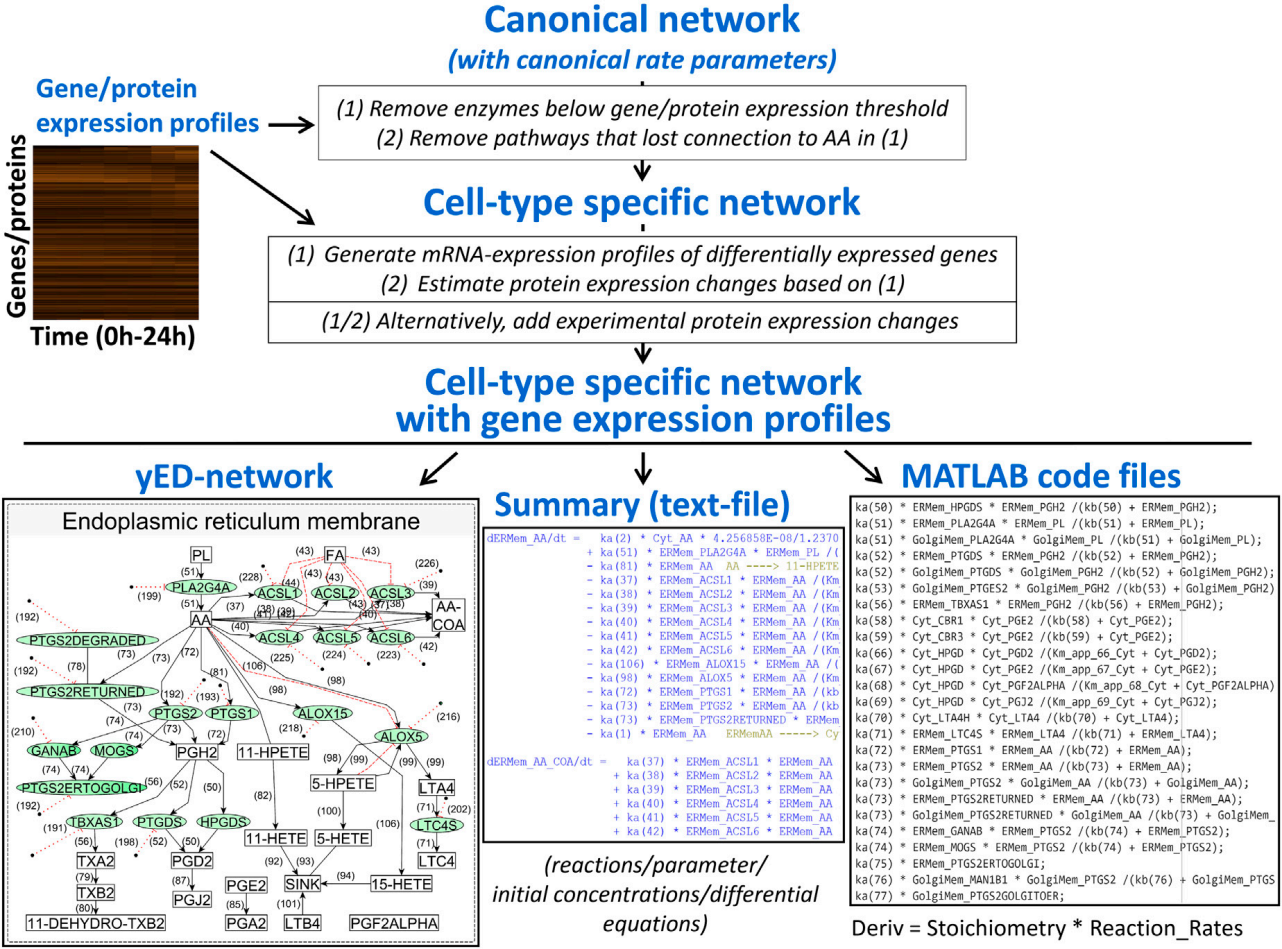
An algorithm that integrates canonical networks of subcellular processes with gene expression profiles to produce cell type specific networks and systems of reactions that can be used for dynamical modeling. AA, arachidonic acid.

We developed a computational pipeline that integrates a canonical model of interest with transcriptomic or proteomic data – either bulk or single cell - to develop cell-type selective dynamical models for the prediction of cell-type selective whole cell responses (Figure 4). The canonical model would involve all known enzymes and reactions described for any cell type of the same organism. Like others (Frohlich et al., 2018), we assume that the reaction rate parameters are canonical as well, i.e., they are the same for all cell types within an organism. Experimental confirmation of our spatial cAMP models (Neves et al., 2008) by others (Castro et al., 2010) indicate that this is likely to be true. Starting sources for the construction of a canonical model could be the KEGG metabolic networks (Kanehisa et al., 2017) or Reactome pathways (Gillespie et al., 2022) for reaction schemas and BRENDA database (Schomburg et al., 2017) for reaction rate parameters. Using transcriptomic and/or proteomic data our computational pipeline adds cell-type selectivity to canonical dynamical models by adjusting steady-state or time-dependent concentrations of enzymes and other proteins to experimentally observed levels.

In more detail, our computational script converts the canonical model into cell-type selective models by first removing enzymes that are not expressed and all reactions that as a consequence lost connection to precursor metabolites because of interrupted substrate flow. In the case of transcriptomic data, our pipeline automatically adds translation and protein degradation reactions to each proteoform in each compartment that can be linked to experimentally determined mRNA levels. Canonical models can be updated based on new knowledge, and our pipeline will generate updated cell-type selective models as well. The individualization of dynamical models from cell-type selective omic datasets has been implemented by other authors as well, studying drug effects on the survival of cancer cell lines (Frohlich et al., 2018). Currently, our algorithm allows compartmentalization of the cell and is capable of predicting metabolite profiles in addition to protein states. After generation of cell-type selective models, our script writes functional MATAB code for each cell type, allowing simulation of cell-type selective responses using standard ODE solvers. Our algorithm can be readily modified to write code for modeling software such as Octave, or Python ODE solvers.

To test our algorithm, we selected arachidonic acid (AA) metabolism that is operative in many cell types and organs. The metabolites generated by this network are important signaling mediators with physiological effects on kidney, uterus and blood vessels as well as other organ systems. Due to the availability of proteomic, transcriptomic and metabolomic datasets from the same experiments, we selected a macrophage cell line, bone-marrow derived macrophages (BMDM), to develop the model and assess its predictive capability.

**FIGURE 5:**
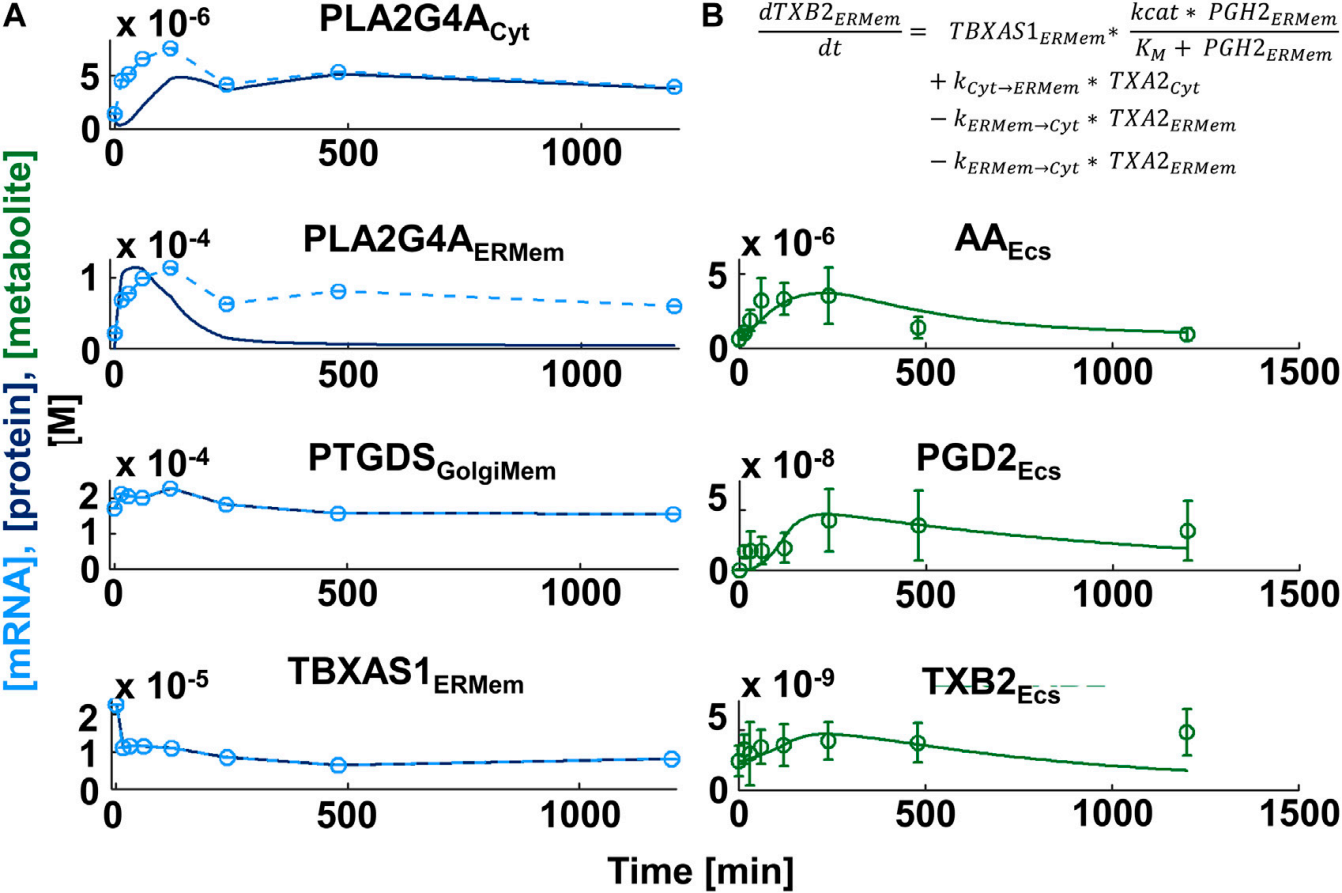
Comparison of simulation and experimental data for production of lipid messengers in macrophages. **(A)** Gene expression profiles (light blue circles in left figure column, n ≥ 3) induced by sequential treatment of Bone-marrow-derived Macrophages with the LPS analogue Lipid A and ATP (Kihara et al., 2014) were mapped to the canonical network of Arachidonic Acid Metabolism and subjected to spline interpolation (light blue dashed lines). Assuming high turnover rates, protein expression time series (dark blue lines) were predicted from mRNA profiles. To allow direct comparison we adjusted the mRNA profile values to lie within the same range as the protein concentrations. ATP stimulation generates a cytoplasmic calcium burst that triggers translocation of multiple eicosanoid enzymes, including cytoplasmic phospholipase A2 (PLA2G4A) from the cytoplasm (Cyt) to intracellular membranes, e.g., the endoplasmic reticulum or Golgi membranes (ERMem, GolgiMem, respectively). Simulated concentrations of the lipid messengers (PGD2 and TXB2 - green lines in right column) agree with lipid messengers measured in the extracellular space (Ecs) (culture medium) in the same experiment (green circles and standard deviations). AA: Arachidonic Acid, PGD2: Prostaglandin D2, TXB2: Thromboxane B2. PTGDS: Prostaglandin D2 synthase, TBXA1: Thromboxane A1 synthase 1. **(B)** Enzyme kinetics in our model are simulated by Michaelis Menton kinetics.

Our canonical model (Figure 4) focused on the synthesis of the major derivatives of AA, i.e., prostaglandins, prostacyclins, thromboxane, leukotrienes and the products of 12- and 15-lipoxygenases (Wang et al., 2021). AA is generated from intracellular membranes by cytosolic phospholipase A2 that is recruited to the site of action by an intracellular calcium peak induced by macrophage activation (Leslie, 2015). Canonical reaction parameters were curated from the literature, if available (PENTACON, 2023). To generate a cell-type-selective dynamic model, we used freely available transcriptomic, proteomic and lipidomic datasets generated from BMDM. The proteomic data described protein expression values in unstimulated BMDMs (Qie et al., 2022) and was used to determine protein expression values at baseline. The published transcriptomic and lipidomic data was generated after BMDM activation by sequential stimulation with Lipid A, an LPS analogue and ATP (Kihara et al., 2014). Both ligands work through cell surface receptors. We used the transcriptomic data to predict how the enzyme expression levels obtained from the proteomic data change in response to macrophage activation. After individualization of the canonical model our script wrote the related MATLAB code that allowed simulation of metabolite profiles after macrophage activation (Figure 5).

The researchers who generated the transcriptomic and lipidomic datasets also published a dynamical model of arachidonic acid metabolism that predicts experimental lipid profiles with high accuracy and showed functional coupling between cyclooxygenases and the terminal synthases (Kihara et al., 2014). We outline the major differences between their and our approaches. These researchers a) simulated reactions using flux dynamics, where fluxes depend on enzyme-specific rate parameters as well as time-dependent enzyme and substrate concentrations. Our equations are based explicitly on Michaelis Menton Kinetics. b) They assumed enzyme protein concentrations follow gene expression values with a delay of 4 h. We use mRNA translation and protein degradation rates to simulate changes of baseline enzyme expression that we predicted from proteomics data. In our model using translation and degradation rates protein expression profiles follow the gene expression profiles with only a short delay. c) The original study focused on the reactions downstream of AA and use the experimental AA time course as a given input for their reactions. Our model includes simulation of AA production and recycling. d) our model contains multiple subcellular compartments, i.e., cytoplasm, endoplasmic reticulum/nuclear membranes, and the Golgi apparatus whose sizes are determined from experimental data. Inclusion of multiple different compartments allows consideration of different intracellular localizations of downstream enzymes (Yuan and Smith, 2015; Calder, 2020), simulation of enzyme membrane recruitments triggered by the calcium peak (Leslie, 2015) as well as vesicular enzyme trafficking (Yuan and Smith, 2015). These realistic details allow for better specification of cell type identity. Overall, our automated algorithm works well (Figure 5). Generally, if initial simulations are substantially different from experimental observations, the model can be revised to add additional cell biological details such as post-translational regulation or additional subcellular compartments. Such variations on a canonical theme model provide a feasible approach to model cell type selective metabolic changes and can be readily adapted to single cell transcriptomic data.

## Dynamical models from single cell transcriptomic data—use of ML-AI approaches

The rapid advances in transcriptomics at the single cell has greatly enhanced our understanding of tissue and organ function. Single cell transcriptomics not only allows us to document the abundances of the different cell types and subtypes in an organ, but also to estimate their capacities for physiological functions. Further, in disease states, single cell transcriptomic measurements enable us to identify infiltrating immune cells and the mechanisms by which they control inflammation and organ responses that can drive disease initiation and progression. Developing accurate computational models of physiological dynamics at the single cell level will be a necessary first step in creating digital twins to understand how organ function changes in disease states. Once an ML or AI algorithm is trained on a particular model, its use can significantly decrease the time needed for simulation with a previously untested sets of expression levels, without loss of quality of the predictions (Nilsson et al., 2022). Such models can also be used to understand the molecular and cellular basis of organ robustness, wherein the organ remains resilient to damage from different types of perturbation including external insults. ML and AI algorithms can also be trained to generate predictions in the opposite direction, i.e., to predict the underlying expression levels from the observed output of the dynamical system. ML and AI algorithms could also help to identify suited drug combinations that generate the desired effect in one cell type, while avoiding the unwanted side effect in another cell type.

Advances in hardware technologies including the development of increasingly fast GPU processers have made the running of thousands to millions of models both cheap and fast. Commercial software such as MATLAB or freeware such as Octave offers programs that that can be used for such simulations. The barriers to using these technologies are mostly at the biological level. The overall biological knowledge of the system being simulated should be utilized to constrain the development of the large-scale simulations with flexibility. Such an approach would prevent simulation of the proverbial spherical cow, but at the same time allow detection of black swans - rare variations in whole cell functions with high impact on physiology.

**FIGURE 6:**
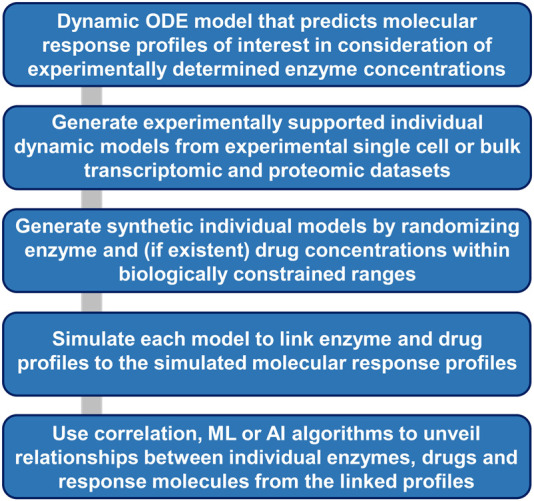
Workflow for ML and AI-based extraction of molecular relationships from simulations of dynamic models. Dynamic models that incorporate experimental or synthetic enzyme concentrations as independent variables allow generation of dependent large-scale simulated response profiles. Statistical, ML and AI algorithms can allow identification of hidden relationships between individual enzyme, drug, and response molecule concentrations.

To fully utilize the knowledge from single cell transcriptomic data, a systematic approach to build organ level dynamical models from single cell transcriptomic data starts with building reasonable models for each cell type and each cell assigned to a cell type (Figure 6). Single cell transcriptomic data indicate that different components of a pathway are expressed at varying levels in individual cells. Model simulations can generate outputs for all observed expression profiles. Additional synthetic training data can be generated by introduction of random variations in enzyme concentrations that lie within biologically reasonable constraints. If the model contains equations describing drug actions, their concentration can be varied in the synthetic and experimental training data using the same rules. Overall, such an approach could allow the generation of thousands or even millions of different models, each of which will link its own enzyme and drug profile to its simulated molecular response profile. Training of ML and AI algorithms on all profiles can unveil relationship patterns between individual molecules or groups of molecules across the three different profiles.

Machine learning approaches are already used as an alternative to classical dynamical modeling for signaling pathways from receptors to transcription factors (Nilsson et al., 2022). Using a genome-scale artificial neural network and synthetic data based on canonical pathways and parameters the model predicted with reasonable accuracy the relationship between ligand receptor interactions and transcription factor activation in macrophages as assessed by transcriptomics.

In other fields that use numerical simulation extensively, neural networks have been successfully used to develop models and make reasonably accurate predictions. Adaptation of graph neural networks that use a “encode-process-decode” approach as described by the authors has been used to develop accurate medium range weather predictions (Lam et al., 2023). This machine learning approach uses network framework where system states (e.g., reactant identity, reactant concentrations) are represented as nodes and dynamics are approximated by message-passing between these nodes. Such systems do not require explicit formulation of the system in terms of differential equations (Sanches-Gonalez et al., 2020), nevertheless are able to learn and produce complex simulations with mesh-based systems (Sanchez-Gonzalez and Battaglia, 2021). Although we have not yet seen the use of such graph neural systems-based models for dynamics from single cell transcriptomics data, it is likely that such simulations will be useful to extract deep knowledge as we accumulate spatial transcriptomic data at the single cell level.

## Cell to tissue models and disease states–integrating with clinical and pathology phenotypes

Cell models as cores of digital twins presume a middle-out format. This format uses a cell centric approach in going from genes to organ level physiological functions. The components (mostly proteins) of pathways and functional units within cells can be connected to genes and their genomic and epigenetic determinants at one end and organ physiology and organismal phenotypes at the other end. Changes in cellular components in different physiological and pathophysiological states are experimentally identified from omics analyses. To make these connections in an explicit manner so distant functional relationships are not only computable but also findable at every scale of organization and traceable across scales we need knowledge graphs that connect components and features both within and between knowledge domains.

**FIGURE 7:**
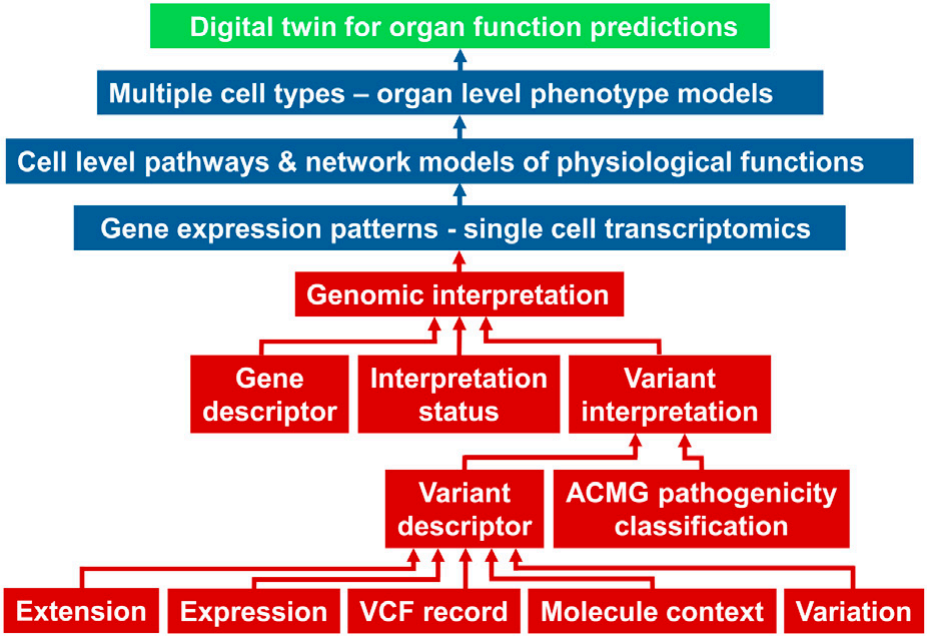
A simplified schema adopted from (Jacobsen et al., 2022) of how genomic descriptors (red boxes) within the Phenopacket schema can be connected to single cell transcriptomic data and models to develop digital twins of organ function. ACMG, American College of Medical Genetics; VCF, variant call format.

An example of framework that connects physiological and pathophysiological characteristics (phenotypes) to genomics at an individual level is the Global Alliance for Genomics and Health (GA4GH) Phenopacket schema (Jacobsen et al., 2022). This schema uses ontology terms across various domains such as genomic variants, pathology, clinical measurements, and therapeutic actions to connect features from one domain to another. Developing Phenopacket-like schemas as knowledge graphs will be the next challenge to be solved to connect cell level physiology to organ phenotypes. In addition to pathways and processes within each cell type at a single cell level, such connections will have to include molecular details of cell-cell interactions and cell-matrix interaction. Technologies advances in spatial transcriptomics, metabolomics, and proteomics, at the single cell level are making it possible to identify and map spatial relationships between individual cells in a single cell type, and between different cell types. A simplified schematic of a digital twin for organ function prediction is shown in Figure 7. The genomic interpretation workflow is taken from the Phenopackets schema (Jacobsen et al., 2022).

Relationships between multicellular structures within an organ such as blood vessels and nephrons in kidney or blood vessels and chambers (e.g., left ventricle) in the heart will be specified in terms of molecular interactions. This spatial knowledge will have to be incorporated into functional models to accurately simulate how cell-level physiology functions enable the emergence of organ phenotypes that are clinically measured, such as estimated glomerular filtration rates for kidney and left ventricular ejection fraction for heart. At the other end of a multilayered knowledge graph, we will have to connect the transcriptomic data in different cell types and subtypes to genomic determinants such as single nucleotide polymorphisms, copy number variations and other features. We will also have to connect epigenetic determinants to transcriptomic profiles. The effects of non-coding RNAs in controlling transcription will have to be mapped to the knowledge graph to fully describe the various modes of regulation that control mRNA levels for translation.

Cell endowment is a concept that emerges from single cell transcriptomics. Cell endowment states that normal function of organ level physiological functions is dependent on the levels of key cell types. Single cell transcriptomic data sets provide information regarding the number of cells in each cell type and subtype in addition to the gene expression profiles and this information will be the basis for important parameters that connect cell physiological events to organ phenotypes. This information can be captured in the knowledge graphs as node attributes at the cell level and used in a quantitative fashion in the numerical models The ability to encode cell endowment within the graph structure is a good example of power of graphs in representing multidimensional biological systems. For such graphs to be properly constructed it is essential that the semantic frameworks within different domains are appropriately and correctly harmonized and that ontology integration is an early focus in development of digital twins.

## Conclusion and perspective

### Challenges in building realistic digital twins for organ function

#### Organ structure

The conversion of cell-level physiology into organ function is in part controlled by the spatial organization of the different cell types within the organ in the context of the extracellular matrix. Additionally, both local and global geometries in the organ will shape biophysical forces that in turn control cell-level physiology through mechanotransduction. Here, we have to account both for the contributions of the extracellular matrix to the overall biomechanical properties of the tissue and organ as well as the interactions of matrix proteins with cell membrane proteins to communicate both biomechanical and biochemical signals to the different cell types. It is likely that these properties will vary from organ to organ and even within regions of an organ. How these similarities and differences are encoded in the knowledge graphs is a challenge that needs to be addressed.

#### Cell biological rules

Physiological functions at the whole cell level are governed by a myriad of rules including those that specify constitutive properties. Such rules need to consider the regulation by the vast signaling networks that transduce external and intracellular signals to control effector functions, such as cytoskeletal dynamics or intracellular degradation pathways. Rules governing the relationship between mRNA and protein levels are of importance as well, when building functional networks from single cell or bulk transcriptomic data. Rules for protein turnover and location are also important and need to be appropriately coded as node attributes. Although there is general concordance between mRNA and protein levels (Buccitelli and Selbach, 2020) this needs to be ascertained for individual proteins of interest and can be done by parameter variation exercises in dynamical models.

Not every cellular function is required for simulation of whole cell physiology that drives organ phenotype. However, for an organ function of interest, it is essential to generate rules on how to simulate the activities of relevant pathways and their functional interactions. For example, for simulating organ functions such as nutrient absorption in the intestines (Kellett et al., 2008), glucose reabsorption in the kidney proximal tubule cells (Chichger et al., 2016) or water reabsorption in the kidney principal cells (Zhao et al., 2023) it is essential that rules governing trafficking (i.e., transport and recycling) of the appropriate transporters, channels and pumps are specified for the cell types of interest. Many of these rules can be generated from the vast experimental literature in cell biology, biochemistry and physiology that have studied individual processes in depth. The rules can be encoded as edge specifications. However, in using prior knowledge, it is important to have strict guidelines in interpreting the experiments to avoid artefactual conclusions. A common example is the caution we need to exercise in extracting rules from studies that overexpress proteins of interest in exogenous systems to obtain insight into native physiological functions.

#### Parameters for interactions

For building dynamical models, obtainment of kinetic parameters for the reactions and concentrations of reactants has remained among the most intractable problems, although databases such as BRENDA (Schomburg et al., 2017) offer great help for this task. Since our early work on bistable switches for cell states in the late nineties (Bhalla and Iyengar, 1999) till today, 25 years later, no systematic effort to develop catalogs of quantitative parameters has been undertaken. This lack of data sets has led us to estimate and guesstimate parameters (Bhalla and Iyengar, 1999; Rangamani et al., 2011) or calculate parameter dependencies (Yadaw et al., 2019) over the years. Others have used the Hill equation approximation (Ryall et al., 2012) which provides biologically relevant simulations as assessed by experiments that test simulation predictions.

Specification of reaction rates is complicated by the fact that often post-translational modifications such as phosphorylation change reaction rates. Hence, these rates need to be specified for different states of the same proteins (proteoforms) (Melani et al., 2022). Additionally, initial concentrations of protein reactants arise from mixtures of these proteoforms and knowledge of the relative proportions of the proteoforms is very valuable in accurately specifying initial concentrations for a group of reactions. Such detailed knowledge exists for very few pathways within the mammalian cell but can be estimated from experimentally obtained overall profiles of pathways activities.

The issues regarding kinetic parameters can lead one to conclude that dynamical models are often not worth the effort. However, this is not so. Dynamical models are important because physiology is dynamics. Unless we can develop and integrate dynamical models with the growing array of informatics and statistical reasoning models we will not achieve the full predictive capability that current large datasets can enable. Artificial intelligence (AI) and machine learning (ML) algorithms that sort through vast arrays of parameter variations in a combinational manner can help. Steady state behavior of stimulated signaling networks has already been successfully modeled with high computational performance using recurrent neuronal networks that reflect network topologies and approximate protein interactions with a perturbation-specific activation function (Nilsson et al., 2022). AI and ML algorithms incorporated at the interface of transcriptomic data derived networks and their casting as dynamical models can help sort through both the rules required to specify and constrain and the parameters needed to run the simulations. Initially such integration will be by trial and error. However, as we develop large libraries of models that predict a range of organ physiological behaviors, we will be able to select well-constrained models for understanding and predicting an organ state or function of interest.

#### Error propagation, uncertainty and accuracy of predictions

The advances in data gathering and enormous growth in computing capability have brought us to the cusp of building accurate computational representations of many organ systems in our body. Integration of the different modeling approaches will ensure that we do not produce spherical cows, rather multiscale models with zoom-in zoom out capabilities where macroscopic functions of the whole organs can be understood and predicted from genomic characteristics underlying molecular and cellular properties. While at 30,000 feet view the ability to develop digital twins that predict organ behavior from genomic information based on mechanistic functions at the molecular and cell level appear achievable given the vast amounts of data in different domains cheap high-performance computing and current advance in machine learning and artificial intelligence algorithms, the picture at the ground level is considerably more complex. There are multiple levels of uncertainty that can lead to propagation of errors resulting in diminishing the accuracy of predictions. At a minimum there are many types of uncertainty 1) within a data domain there can be uncertainty regarding node size and attributes 2) within molecular interaction domains uncertainty regarding the existence of edge and edge strength 3) uncertainty in connections between edges and potential interdomain edges being affected by distal domains. 4) errors in computations arising from methods of simulations, such as errors due to large time steps in ODE models. There is a need to develop methods to quantify each of these uncertainties and error generating steps and develop an overall numerical score that reflects the reliability and accuracy of prediction. It is likely this will be a separate sub-field in the development of digital twins for organs.

It is commonly understood that each individual is different from others, but nevertheless belongs to groups or categories of physiological functions such that disease states in these groups can be treated with similar therapeutic approaches. It is also commonly observed in clinical practice that some individuals within a therapeutically defined group need to have a personalized therapeutic strategy that is optimal to control their pathophysiology. Currently this is done empirically by trial and error. As accurate digital twins are developed, we should be able to predict the clinical responses of these individuals for optimal therapeutic benefits.

## Data Availability

Publicly available datasets were analyzed in this study. This data can be found here: Kihara et al. (2014) and Qui et al. (2022).

## Code availability statement

Modeling code for the reproduction of the simulations shown in Figure 5 can be found at ‘github.com/SBCNY/Integration_transcriptomics_dynamicModels’ in the folder ‘Arachidonic_acid_metabolism’.
